# Tumour suppressor gene methylation and cervical cell folate concentration are determinants of high-risk human papillomavirus persistence: a nested case control study

**DOI:** 10.1186/1471-2407-14-803

**Published:** 2014-11-03

**Authors:** Janet E Flatley, Alexandra Sargent, Henry C Kitchener, Jean M Russell, Hilary J Powers

**Affiliations:** Human Nutrition Unit, Department of Oncology, Faculty of Medicine, Dentistry and Health, University of Sheffield, Sheffield, S10 2TN UK; Department of Clinical Virology, Central Manchester University Hospitals, Manchester, M139WL UK; Gynecological Oncology Group, Cancer Studies, Faculty of Human and Medical Sciences, University of Manchester, Manchester, M13 9BL UK; Corporate Information and Computing Services, University of Sheffield, Sheffield, S10 2FN UK; Human Nutrition Unit, Department of Oncology, Faculty of Medicine, Dentistry and Health, University of Sheffield, Sheffield, S10 2TN UK

**Keywords:** Folate, DAPK methylation, HPV persistence, Cervical cancer

## Abstract

**Background:**

Persistent infection with one or more high-risk human papillomavirus [HR-HPV] types increases the risk of intraepithelial neoplasia and cervical cancer. A nested case–control study was conducted to investigate the importance of cervical cell folate concentration and tumour suppressor gene methylation as risk factors for HR-HPV persistence.

**Methods:**

Cervical cell samples from 955 women with HR-HPV infection and normal, borderline or mild dyskaryosis were retrieved from the archive of a population-based screening trial. Women were classified as cases or controls, reflecting the presence or absence [respectively] of any HR-HPV infection at a follow-up clinic at least 6 months from baseline. Cervical cell folate concentration and promoter methylation of five tumour suppressor genes were measured in independent samples from cases and controls.

**Results:**

A higher cervical cell folate concentration [P = 0.015] was an independent predictor of infection at follow-up, together with infection with HPV-16 or infection with multiple HR-HPV types. Methylation of the tumour suppressor gene *DAPK* was associated with a 2.64-fold [95% CI, 1.35-5.17] increased likelihood of HPV infection whilst *CDH1* methylation was associated with a 0.53-fold [95% CI, 0.331-0.844] likelihood of HR-HPV infection at follow-up. When considering women with normal or abnormal cytology, the predictive effect of higher cervical cell folate was only seen in women with mild cytology [P = 0.021]; similarly the effect of *DAPK* methylation was seen in women with mild or borderline cytology [P < 0.05].

**Conclusions:**

Higher cervical cell folate concentration and promoter methylation of the tumour suppressor gene, *DAPK*, in women with cervical cell dyskaryosis, are associated with increased risk of HR-HPV persistence.

## Background

Infection with one or more high-risk human papillomavirus [HR-HPV] types increases the risk of the occurrence and progression of cervical intraepithelial neoplastic [CIN] lesions and invasive cervical cancer [1]. Whilst the majority of infections are transient and resolve of their own accord, a recent meta-analysis gave a summary estimate for HR-HPV persistence [HR-HPV positive at 2 or more consecutive time-points] of about 40%, for both nontype-specific and type-specific HR-HPV [2].Women with persistent infection with HR-HPV have a greater risk of developing cervical cancer; the risk is greater for those with type-specific persistence [3]. A number of factors are thought to influence the likelihood of HR-HPV persistence, including immunocompetence, use of oral contraceptives, smoking, parity, genotype and diet, although results are not wholly consistent [2, 4–6].

There is evidence that folate status influences the natural history of HPV infection. A prospective follow-up study that monitored 345 women over a 24-month period showed that women with low folate status were at a higher risk of acquiring a HR-HPV infection and of repeat infection with HR-HPV, than women with higher folate status [7]. Furthermore, in our previous cross sectional study of 308 women with different cervical cytology, women with HR-HPV infection had a lower red blood cell folate concentration than women free of infection [P <0.05] [8]. In the same study it was shown that women diagnosed with CIN grades 1, 2, or 3, or cancer had a significantly lower red cell folate status than those with normal cervical histology, independent of HPV status [P < 0.05] [8].

The usefulness of tumour suppressor gene hypermethylation as a prognostic biomarker is under intense investigation in many different cancers, including cervical cancer and its precursor lesions. Several groups, including our own, have reported that high-grade cervical cell abnormality or invasive cervical cancer is associated with an increased likelihood of promoter methylation of selected tumour suppressor genes compared with normal cells [8–10]. Folate, as a methyl donor, is considered to be an important determinant of normal DNA methylation, although direct evidence that folate status influences gene specific methylation in a predictable and consistent manner in humans is lacking.

The approach to cervical cancer prevention up until now has involved primary screening for cytological abnormalities followed by direct referral to colposcopy for women with persistent borderline changes, mild, moderate and severe dyskaryosis. HPV triage is now being implemented across England, which involves HPV testing in the event of cytology reported as borderline or mild dyskaryosis. Women who are HPV positive are referred to colposcopy and HPV negative women are referred back to routine recall. At colposcopy women with < CIN1 are not treated and referred back to routine recall whilst women with CIN1 have a repeat cytology and HPV test at 6 months [11]. Predictive markers of HPV persistence may be clinically useful and inform patient management.

We conducted a case–control study, nested in a randomised trial [ARTISTIC; *A R*andomised *T*rial *I*n *S*creening *T*o *I*mprove *C*ytology] conducted within the routine NHS Cervical Screening Programme in Greater Manchester [12], to assess the predictive power of cervical cell folate status and tumour suppressor gene methylation in determining HR-HPV clearance.

## Methods

### Study design and sample retrieval

A nested case–control study was conducted within the ARTISTIC study. Ethics approval was obtained from Multicentre Research Ethics Committee, North West, UK [MREC 00/8/30]. The ARTISTIC study was primarily concerned with examining the potential value of HPV detection in cervical cell samples, to enhance screening for cervical cancer risk [13]. Women were randomised to the HPV result being revealed or being concealed. An archive of over 20,000 liquid-based cervical cell samples collected from women aged 20–64 years old at baseline and at follow-up appointments was established [12]. Samples from all women with normal, borderline or mild cell dyskaryosis, representing the range from normal to early changes in cervical cytology, were selected from the ARTISTIC archive for this nested case–control study. This group comprised 955 women. Samples [n = 482] were selected from women who were HR-HPV positive at baseline and positive for any HR-HPV at a follow-up clinic at least 6 months later; these women were assigned ‘case’ status. Samples [n = 473] from women diagnosed as HR-HPV positive at baseline, but HR-HPV negative at a follow-up clinic at least 6 months later were assigned ‘control’ status. For the purpose of this study, HPV persistence was defined as infection with any HR-HPV type at follow-up. In the ARTISTIC study HPV genotyping was carried out using the Roche reverse line blot assay [13]. Samples were collected from the Manchester archive in batches and transported, frozen, to Sheffield, for storage at -80°C.

Samples were randomly assigned to gene methylation or folate measurement. Previous experience had indicated that a single cervical cell sample from one woman would generally provide insufficient material for the reliable measurement of cervical cell folate concentration. The pooling of biological samples is an accepted means of overcoming analytical limitations imposed by small sample volumes [14, 15]. It had been anticipated that at least two samples would need to be pooled for the measurement of cervical cell folate, therefore 556 samples were selected for folate measurements and 399 samples were selected for gene methylation measurements.

### Cervical cell folate

In total, 556 cervical cell samples [cases n = 283; controls n = 273] were analysed for the concentration of total folates. Three samples were pooled from those with normal cytology, 2 samples were pooled from those with borderline or mild cytology. Pooling was random within cytology groups, for both cases and controls. Cervical cell samples, each suspended in 250 μl PBS, were pooled, and cell lysis performed using the Precellys^®^24 homogenizer using the CK14 ceramic bead kit [Bertin Technologies]. Folate concentration was measured using the Access folate competitive binding assay; the protein concentration of the supernatant was analysed using a protein assay [Bio-Rad] and folate concentration was expressed as ng/mg protein. A total of 217 measurements of tissue folate were made, reflecting an average pooling of 2.5 samples.

### DNA hypermethylation

Of 399 samples analysed for gene-specific methylation, 199 were cases and 200 were controls. Promoter methylation of some tumour suppressor genes has been shown to be greater in cervical cell dysplasia, and cervical cancer, than in normal cervical tissue and we have previously postulated that a gene-specific hypermethylation profile might be used as a predictive biomarker of cervical cancer risk [8]. Additionally, methylation of the HPV genome can influence virus activity in the host cell [16]. Five tumour suppressor genes were selected for study on the basis of consistent evidence for hypermethylation in cervical dysplasia or cancer [17]. These were, *DAPK, CDH1, MGMT, MLH1* and *p16*. Genomic DNA was isolated from cervical cells using the QIAmp mini DNA kit [Qiagen] according to manufacturer’s instructions and quantified using the Nanodrop ND-1000. For DNA methylation analysis, 2 μg of genomic DNA were modified with a sodium bisulphite treatment as previously described [8]. Quantitative methylation-specific PCR [QMS-PCR] was used to determine the CpG methylation status of the five tumour suppressor genes, and of β-actin as an internal reference gene, using the ABI StepOnePlus™ system. Primer sets and TaqMan probes for *ACTB, DAPK, CDH1, MLH1* [18], *MGMT* [19] and *p16* [20] were obtained from Sigma-Genosys and Applied Biosystems respectively. Placental DNA [Sigma], methylated using CpG methyltransferase [M.SssI] [New England Biolabs] and sodium bisulphite treated, was used as a positive methylated control in each assay. A negative no-DNA-template control was also included in every run. The assay was performed in a reaction volume of 20 μl in 96 well plates. The final reaction volume was composed of 1 X TaqMan^®^ Fast Universal PCR master mix, no AmpErase^®^ UNG [Applied Biosystems], 4 pmol of each primer, 2 pmol TaqMan probe, 10 ng of template and water. PCR was performed under the following conditions: denaturing at 95°C for 20 s followed by 40 cycles of 95°C for 1 s and 60°C for 20 s.

The PCR efficiencies of the target and reference gene (ACTB) were checked and found to be within 10% of one another and considered valid for the delta Ct calculation. The Ct threshold was determined automatically by the ABI software. Each sample was analysed in triplicate and the result was only considered valid if 2 out of 3 triplicates, and the positive control, showed amplification above the set Ct threshold. PMR was calculated as previously described [21]: [target gene:ACTB sample /target gene:ACTB ratio in control methylated DNA] x 100.

We had anticipated low levels of DNA methylation, if present at all, in cell samples with low level cytological abnormality [8]. On this basis we classified the detection of any methylation as being methylated so if PMR > 0 the sample was classed as being methylated.

Ct values for ACTB for the methylation control sample were compared across sample runs within assays for individual target genes and across assays for the 5 different target genes as a measure of assay precision. Coefficients of variation (CV%) were as follows: *DAPK* 2.9%, *CDH1* 2.8%, *MGMT* 2.6%, *MLH1* 2.9%, *P16* 2.8%.

### Statistical analysis

Mann Whitney U test was used to compare age between women in case and control groups and between folate and methylation subsets within case and control groups. A comparison of the prevalence of individual HPV types between cases and control groups was made using the Chi-squared test. Cervical cell folate concentration was compared between cases and controls, and between women with normal and abnormal cytology, using the Mann Whitney U test. Logistic regression analysis was used on the folate subset to examine the importance of age, HR-HPV strain, and folate concentration as independent predictors of HR-HPV clearance and on the gene methylation subset to examine the importance of age, HR-HPV type and tumour suppressor gene methylation as independent predictors of HR-HPV clearance. The Benjamini-Hochburg correction was used to correct for overfitting in multivariate testing. P < 0.05 was taken to indicate statistical significance.

## Results

The main focus of interest was factors which discriminated women who were observed to carry an HR-HPV infection at a follow-up clinic [cases] from those who did not [controls].

### The total cohort

Women in the case group had a mean [SD] age of 30.05 [8.92] years, those in the control group had a mean [SD] age of 31.56 [9.49] years [Table 1]. Table 1 also shows mean ages of women in the folate and methylation subsets. There were no differences in age between cases and controls, for the total sample or for the two subsets, or between folate and methylation subsets within cases or controls.

**Table 1:** **Age of women in the study, according to case or control status**

	Cases		Controls	
	n	age [y]	n	age [y]
*Total cohort*	482	30.05 ± 8.92	473	31.56 ± 9.49
*Folate cohort*	283	30.51 ± 9.46	273	30.10 ± 8.46
*Methylation cohort*	199	29.39 ± 8.08	200	33.56 ± 10.43

Values are presented as means ± SD, for women in the total cohort, and the folate and methylation sub-groups, according to case or control status.

Table 2 shows the prevalence of infection with specific high-risk HPV types for the whole cohort, according to case or control status and cytology. The most prevalent infection was HPV-16, with a prevalence of 25%. Of those HPV types showing an overall prevalence greater than 10%, HPV-16 and -52 were more prevalent in cases than controls [P < 0.02]; strain 56 was less prevalent in cases than controls [P < 0.05]. 50% of women were infected with more than one HPV type.

**Table 2:** **Baseline HPV prevalence [%] for total cohort according to case–control status and cytology**

HPV type		Cases				Controls		
	*Normal*	*Borderline*	*Mild*	*All*	*Normal*	*Borderline*	*Mild*	*All*
16	29.3	23.0	32.3	28.6^a^	20.5	23.1	25.3	22.0
18	14.8	19.2	13.13	15.4	13.1	13.3	6.1	11.6
31	11.7	17.3	14.14	13.3	9.5	11.0	2.0	8.3
33	5.0	11.0	8.08	6.9	5.7	12.1	2.0	6.1
35	4.2	6.1	4.04	4.6	3.5	6.6	6.1	4.7
39	9.8	11.0	10.10	10.2	9.2	13.2	13.1	10.8
45	8.1	2.7	9.09	7.1	8.5	5.5	5.1	7.2
51	11.0	12.1	15.15	12.0	9.9	7.7	28.3	13.3
52	18.4	13.0	18.18	17.2^a^	9.9	22.0	9.1	12.1
56	4.2	5.3	12.12	6.0	8.8	12.1	12.1	10.2^b^
58	5.0	11.2	17.17	8.7	5.7	3.3	3.0	4.7
59	10.6	6.1	4.04	8.3	7.1	5.5	5.1	6.3
68	3.9	2.6	6.06	3.9	4.6	1.1	5.1	4.0
73	3.5	2.0	2.02	2.9	2.5	4.4	4.0	3.2
82	0.40	1.1	0	0.4	2.8	0.0	0.00	1.7
*N*	*283*	*100*	*99*	*482*	*283*	*91*	*99*	*473*

^a^significantly greater than controls, P < 0.02; ^b^significantly greater than cases, P < 0.05.

### Folate subset

The folate subset was comparable with the whole cohort in terms of age and HPV infection profile. Cervical cell folate concentration was higher in women with normal cytology [median 3.41, 25th centile 1.94, 75th centile 6.72 ng/mg protein] than those with borderline or mild cervical cell abnormality [median 2.68 ng/mg protein, 25th centile 1.22, 75th centile 6.11] [P = 0.002] [Table 3]. The table also shows cervical cell folate concentration according to case and control status, for each cytology group. In women with the most abnormal cytology [mild], the cervical cell folate concentration was significantly higher in cases [median 3.88 25th centile 2.16, 75th centile 6.94] than controls [median 2.33, 25th centile 1.02, 75th centile 3.55] [P = 0.004].

**Table 3:** **Cervical cell folate concentrations [ng/mg protein] according to case or control status, and cytology**

		Normal cytology	Borderline abnormality	Mild abnormality	Mild + Borderline	All
**Cases**	*n*	61	25	25	50	111
	*median*	3.23	2.28	3.88**	3.11	3.21
	*interquartile range*	1.88-7.08	0.73-6.11	2.16-6.94	1.00-6.77	1.80-7.03
**Controls**	*n*	61	20	25	45	106
	*median*	3.60	2.97	2.33	2.50	3.03
	*interquartile range*	2.30-6.00	2.12-4.89	1.02-3.55	1.47-4.10	1.83-5.42
**All**	*n*	122	45	50	95	217
	*median*	3.41	2.68	2.72	2.68*	3.07
	*interquartile range*	1.94-6.72	0.92-6.11	1.36-5.82	1.22-6.11	1.81-6.21

A total of 556 cervical cell samples were used for folate measurements, 2–3 samples were pooled for these measurements, n values refer to the number of actual measurements made.

*Significantly lower than in women with normal cytology [*P* = 0.002].

**Significantly higher than in controls [*P* = 0.004].

Logistic regression analysis of determinants of HR-HPV infection at follow-up was initially carried out on the whole folate subset; age, cervical cell folate concentration, infection with HPV-16, -18, 52, and infection with multiple HPV types, were entered into a step-down model. In the whole sub-set, higher cervical cell folate concentration [P = 0.015], infection with HPV-16 [P = 0.038], or infection with more than one HR-HPV type [P = 0.038], were each independently and significantly predictive of a HR-HPV infection at follow-up. When however the analysis was conducted in different cytology groups, other determinants were identified. In women with normal cytology at baseline, the age, the presence of HPV-16 infection and the presence of multiple infections were all significantly predictive of being a case rather than a control [Table 4]. Effects are multiplicative, meaning that, for example if we compare two women with normal cytology, one woman who is aged 20 and has no HPV-16 infection, the other woman who is 30 and has multiple infections including HPV-16, this latter woman has a 415% increased risk of having an HR-HPV infection at follow-up. Among women with mild abnormalities at baseline [Table 3], a lower age, a higher cervical cell folate concentration, and infection with HPV-52 were all significantly predictive of being a case rather than a control. For women with this level of cervical cell abnormality, for every ng folate/mg protein increase in cervical cell folate concentration, a woman would be 14.4% more likely to have an HR-HPV infection persist. No significant determinants of HR-HPV persistence emerged for women with a diagnosis of borderline abnormality. When the analysis was repeated for the borderline and mild cytology groups combined, the final model showed that lower age [P = 0.027], [OR 0.959, CI 0.923, 0.996] and higher folate concentration [P = 0.013], [OR 1.099, CI, 1.107-1.187], were significantly predictive of HR-HPV infection persisting.

**Table 4:** **Determinants of HR-HPV persistence for women with normal or mild cervical abnormality, for the folate sub-set; results of a multivariate analysis**

Variable	Odds ratio [95% CI]	P value
*Total sample*		
Folate [ng/mg]	1.065 [1.012; 1.120]	0.015
HPV-16 infection	1.509 [1.022; 2.228]	0.038
Multiple HR-HPV infections	1.440 [1.026; 2.019]	0.036
*Normal cervical cytology*		
Age	1.031 [1.007; 1.055]	0.012
HPV-16 infection	1.915 [1.176; 3.119]	0.009
Multiple HR-HPV infections	1.570 [1.020; 2.416]	0.040
*Mild cervical cytology*		
Age	0.925 [0.872; 0.982]	0.010
Folate [ng/mg]	1.144 [1.020; 1.282]	0.021
HPV-52 infections	4.924 [1.310; 18.513]	0.018

HPV persistence refers to any HR-HPV infection at follow-up.

### Methylation subset

**Figure 1:**
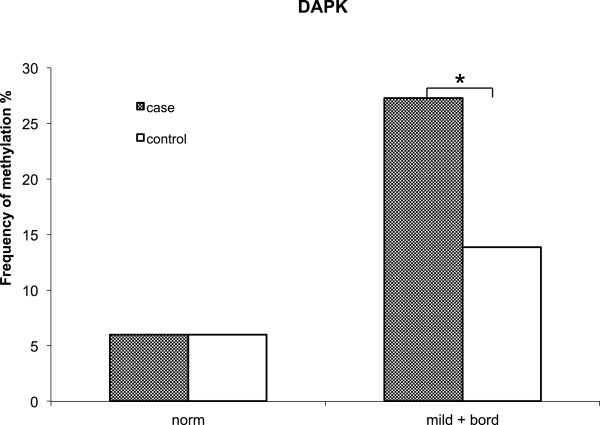
**Frequency of** ***DAPK*** **methylation according to case or control status and cytology.** Gene promoter methylation determined in cervical cells from 199 cases and 200 controls. Comparison between cases and controls in women with normal cytology [norm] or with borderline or mild [mild + bord] cellular abnormality. *P < 0.05 significantly greater in cases than controls.

Tumour suppressor gene methylation was examined in a subset of 399 women. This subset was comparable to the whole cohort in terms of age and frequency of infection with specific HPV strains. Prevalence of specific HR-HPV strains was comparable with the folate subset; 25% of the women were infected with HPV-16 and 10 - 16% of women carried an infection with HPV-18, -31, -39, -51 or -52**.** Table 5 shows the methylation data for this subset. *CDH1* had a relatively high frequency of methylation [up to 56%], particularly in the samples with mild and borderline cytology. The frequency of *DAPK* methylation was low in women with normal cytology, both for cases and controls, compared with women with borderline or mild cytology. The methylation of *MGMT* gene was similar [10-14%] for all levels of cytology and cases and controls groups. A low frequency of methylation for *p16* and *MLH1* was detected across all cytological groups in both cases and controls. Logistic regression analysis for the methylation subset as a whole showed that infection with HPV-18 [P = 0.028], -52 [P = 0.007] or -58 [P <0.001] and promoter methylation of *DAPK* or *CDH1* [P < 0.01] were predictive of HR-HPV infection at follow-up. Methylation of *DAPK* was associated with a 2.64-fold [95% CI, 1.35-5.17] increased likelihood of HR-HPV infection at follow-up whilst *CDH1* methylation was associated with a 0.53-fold [95% CI, 0.331-0.844] likelihood of HR-HPV infection at follow-up. Data were also examined according to whether women had normal or abnormal cytology [borderline and mild combined] at baseline. In women with borderline or mild cervical cell abnormality at baseline, but not in women with normal cervical cells, DAPK had a significantly higher frequency of methylation in cases than controls [Figure 1] [P < 0.05].

**Table 5:** **Frequency of gene specific methylation according to case or control status and cytology group**

	*Frequency of sample methylation [%]*									
	DAPK		CDH1		MGMT		MLH1		P16	
	Case	Control	Case	Control	Case	Control	Case	Control	Case	Control
*Mild*	26.5 *[13/49]*	14 *[7/50]*	32.7 *[16/49]*	40 *[20/50]*	12.2 *[6/49]*	10 *[5/50]*	0 *[0/49]*	0 *[0/50]*	2.0 *[1/49]*	0 *[0/50]*
*Borderline*	28 *[14/50]*	14 *[7/50]*	42 *[21/50]*	56 *[28/50]*	14 *[7/50]*	10 *[5/50]*	0 *[0/50]*	0 *[0/50]*	0 *[0/50]*	0 *[0/50]*
*Normal*	6 *[6/100]*	6 *[6/100]*	26 *[26/100]*	33 *[33/100]*	10 *[10/100]*	10 *[10/100]*	1 *[1/100]*	0 *[0/100]*	5 *[5/100]*	7 *[7/100]*
*Total*	23.6^a^ *[47/199]*	17 *[34/200]*	31.7 *[63/199]*	40.5^b^ *[81/200]*	11.6 *[23/199]*	5.5 *[11/200]*	0.05 *[1/199]*	0 *[0/200]*	3 *[6/199]*	3.5 *[7/200]*

Promoter methylation of selected tumour suppressor genes, expressed as a percentage of women in each group. Values are shown for cases and controls, according to cytology group.

^a^Significantly greater than controls, P = 0.001; ^b^Significantly greater than cases, P = 0.016.

## Discussion

Persistent infection with high-risk HPV increases the risk of cervical cancer. In this study, cervical cell folate concentration, tumour suppressor gene methylation, and particular HR-HPV types, were all shown to be associated with an increased likelihood of persistent HR-HPV infection, defined as infection with any HR-HPV type at follow-up. HPV persistence has been defined and estimated in a number of ways; a recent meta-analysis providing data on more than 100,000 women worldwide, found that 73% of studies defined persistence as HPV positivity at a minimum of two time points [2].

Consistent with the literature, HPV-16 was the most prevalent infection [22]. HPV-16 infection and multiple HR-HPV infections were found to be significant independent determinants of persistent HR-HPV infection, which is consistent with findings from a study of type-specific HPV persistence in Finnish women [23].

Cervical cell folate concentration was higher in women with normal cytology than women with borderline or mild cytology, which is consistent with previous findings in which folate was measured in red blood cells [8] or serum [24]. Multivariate modelling, taking account of HR-HPV-type, infection with multiple HR-HPV types, and a woman’s age, showed higher cervical cell folate concentration to be associated with an increased likelihood of HR-HPV persistence. When the analysis was conducted in each of the three cytology groups separately, this effect was only evident in women who had mild cervical cell abnormalities.

Few other studies on folate status in cervical tissue have been published. The concentrations of folate in cervical cells collected by liquid-based cytology in this study were comparable to those measured in cervical biopsies by Fowler et al., who reported concentrations of 2.75 to 4.39 ng/mg protein [25]. In their small cross-sectional study, Fowler et al. reported a higher mean concentration of cervical cell folate in women who tested HR-HPV positive [4.05 ng/mg] than in women who tested HR-HPV negative [3.13 ng/mg], but the difference did not reach statistical significance. In contrast, Flatley et al. [8] found a lower red blood cell folate concentration in women carrying an HR-HPV infection compared with those free of infection and Piyathilake et al. [7] showed that a higher circulating folate concentration was associated with a greater likelihood of clearing an HR-HPV infection. Unlike Piyathilake et al. (7) we were able to examine a possible role of folate in HPV persistence taking cytology into account as well as HR-HPV type and infection with multiple HPV types. This is important because the complex literature around folate and cancer suggests very strongly that the presence of cell abnormality influences the association. Generally, studies have suggested that a better folate status might protect against certain cancers but that where an underlying neoplasm exists, supplemental folate might accelerate carcinogenesis [26, 27].

Finding from this study differ from the Piyathilake study (7) in which folate was measured in the blood. It is not clear whether the concentration of folate in the blood is strongly correlated with that in cervical tissue, particularly in non-normal tissue. Some cancer cells are known to upregulate folate receptors [28], which facilitates folate uptake and could fuel enhanced cell proliferation. Although cervical cancer cells (HeLa cells) do express a high density of the folic acid receptor [29], it is not known whether this represents an upregulation from the normal cell. An immunohistochemical study of folate receptor expression in cervical tissue showed no difference between normal cells and low-grade abnormalities and a reduced expression in higher-grade abnormalities and cancer [30]. In our study, the higher concentration of folate in the cases would be expected to increase host cell proliferation and this would facilitate viral replication. The downstream effect on persistence is not clear; enhanced viral replication might lead to greater re-infection of adjacent sites, but it might also lead to adaptive immune responses. A recent elegant study of HPV integration in human keratinocytes showed that folate deficiency impaired the cells’ ability to make HPV-16 virion particles, and this was associated with enhanced integration into the host DNA [31]. In our study, cells with higher folate concentration may have been able to produce more virus particles than cells with lower folate concentration, and therefore might have been more permissive of re-infection.

The temporal relationship might be between a lower folate concentration and low-grade cell abnormalities, as observed in the ‘baseline’ measurements is not known. Neither can we be certain what the temporal relationship between a persistent HPV infection and cell folate concentration might be. Although we have discussed how a higher folate concentration might increase the likelihood of HR-HPV persistence, given the understanding that HPV infection can lead to changes in DNA methylation and thereby alter expression of genes [32], we cannot rule out the possibility that persistent HPV infection and cell changes have a synergistic influence on the expression of the folate receptor and folate uptake into cells.

In the multivariate analysis, the association between age and risk of HPV persistence was different, depending on the underlying cytology. In the total sample, age was not associated with risk of HPV persistence. We did not have access to demographic data on alcohol and tobacco use for women whose samples were used in this study; it is possible that there was a difference between younger and older women which could have confounded the association between age and HPV persistence. Cigarette smoking and alcohol consumption are both thought to influence HPV infection and the risk of cervical cancer and both show an age association. Studies have examined HR-HPV persistence by age but the meta-analysis of HR-HPV persistence by Rositch et al. [2] reports no consistent trend.

Promoter methylation of the tumour suppressor gene, death-associated protein kinase, *DAPK*, was also associated with an increased likelihood of HR-HPV persistence. *DAPK* has a known role as a promoter of programmed cell death and *DAPK* promoter methylation has been reported for several cancers including cervical cancer [33, 34]. Promoter methylation of this gene is associated with gene silencing [35]. A link with HR-HPV persistence has not been reported previously but there is a plausible mechanism for a causal relationship. Viruses have evolved different strategies to avoid the host immune response to infection. The inhibition of apoptosis is important to viral pathogenesis. Should *DAPK* be silenced through promoter methylation it no longer promotes cell death via the normal apoptotic pathway of an HR-HPV-infected cell and the host cell may survive and differentiate. Under such circumstances the HR-HPV virus would have longer to replicate, increasing copy number and the likelihood of infecting other cells. A mechanistic link between *CDH1* methylation and HR-HPV persistence is less easy to explain. Promoter methylation of this gene can lead to gene silencing [9]. *CDH1* is a member of the cadherin family, loss of expression would be expected to reduce cell-cell contact, leading to an increase in cell motility and invasion, hallmarks of metastasis. Whilst cadherin expression is important to bacterial adherence and internalisation, this group of proteins has not been implicated in cellular uptake of viruses, although cellular uptake by endocytosis is common to both bacteria and viruses [36].

The temporal relationship between gene methylation, cervical cell folate concentration and infection with HR-HPV in baseline samples is not clear. HR-HPV infection can induce change in gene methylation [37]. This would require recruitment of the host cell methylation apparatus and utilisation of intracellular folate, as methyl donor. Folate status of the cell may influence DNA methylation through effects on DNA methyltransferases [DNMTs]. DNMT downregulation in response to folate depletion has been reported for human colon cancer cells in vitro [38] and unpublished data from our laboratory show that methyl donor depletion of cervical cancer cells *in vitro* leads to downregulation of DNA methyltransferases. By inference, higher cellular folate might increase DNMT expression, and facilitate *DAPK* methylation. This provides a putative link between higher cell folate status and *DAPK* methylation in HR-HPV infection. The low frequency of *DAPK* methylation in women with normal cytology is compatible with our previous findings [8] and other studies showing that *DAPK* methylation occurs on the pathway of HR-HPV-induced cell transformation [10] may explain the lack of association with HR-HPV persistence in women with normal cytology.

It would have been preferable to have had access to sufficient cervical cell material to allow the measurement of tissue folate concentration and gene methylation on the same samples, and to avoid sample pooling for folate measurements. This must be considered a limitation of the study but is unlikely to be resolved without access to biopsy material. It would also have been useful to have been able to include information about smoking and alcohol use into the multivariate analyses, as these are factors are thought to influence the process of viral infection and clearance.

## Conclusions

Persistent infection with HR-HPV causes cervical cancer, and therefore factors which influence the natural history of HR-HPV infection may be important modulators of cervical cancer risk, but the mechanisms that favour HPV persistence are not understood. We have shown that a higher concentration of folate in cervical cells, and promoter methylation of the tumour suppressor gene *DAPK*, in women with cervical cell dyskaryosis, are associated with increased risk of HR-HPV persistence.

We hypothesize that HR-HPV infection induces *DAPK* methylation in dyskaryotic cells, supported by a high intracellular folate, and that *DAPK* methylation leads to dysregulation of apoptosis and promotes HR-HPV persistence. There is a need for *in vitro studies* to examine these hypotheses and so shed further light on mechanisms of viral persistence. An understanding of such mechanisms may have predictive value and inform patient management.

## Abbreviations

HR-HPV: High-risk human papillomavirus
CIN: Cervical intraepithelial neoplasia
ARTISTIC: *A R*andomised *T*rial *I*n *S*creening *T*o *I*mprove *C*ytology
*DAPK*: Death–associated protein kinase
CDH1: Cadherin-1
MLH1: MutL homolog1, colon cancer, nonpolyposis type2 (E. coli)
MGMT: 0-6-methylguanine-DNA-methyl transferase
p16: Cyclin-dependent kinase inhibitor 2A
ACTB: Beta-actin
PMR: Percent methylation reference.
